# Real-Time Monitoring of Infectious Disease Outbreaks with a Combination of Google Trends Search Results and the Moving Epidemic Method: A Respiratory Syncytial Virus Case Study

**DOI:** 10.3390/tropicalmed8020075

**Published:** 2023-01-19

**Authors:** Dawei Wang, Andrea Guerra, Frederick Wittke, John Cameron Lang, Kevin Bakker, Andrew W. Lee, Lyn Finelli, Yao-Hsuan Chen

**Affiliations:** 1 Health Economic and Decision Sciences, Merck & Co., Inc., Kenilworth, NJ 07065, USA; 2 Clinical Development, MSD, Kings Cross, London EC2M 6UR, UK; 3 Clinical Development, MSD, CH-6005 Luzern, Switzerland; 4 Clinical Development, Merck & Co., Inc., Kenilworth, NJ 07065, USA; 5 Health Economic and Decision Sciences, MSD, Kings Cross, London EC2M 6UR, UK

**Keywords:** RSV, Google Trends, epidemiology, Moving Epidemic Method, nowcast, real-time

## Abstract

The COVID-19 pandemic has disrupted the seasonal patterns of several infectious diseases. Understanding when and where an outbreak may occur is vital for public health planning and response. We usually rely on well-functioning surveillance systems to monitor epidemic outbreaks. However, not all countries have a well-functioning surveillance system in place, or at least not for the pathogen in question. We utilized Google Trends search results for RSV-related keywords to identify outbreaks. We evaluated the strength of the Pearson correlation coefficient between clinical surveillance data and online search data and applied the Moving Epidemic Method (MEM) to identify country-specific epidemic thresholds. Additionally, we established pseudo-RSV surveillance systems, enabling internal stakeholders to obtain insights on the speed and risk of any emerging RSV outbreaks in countries with imprecise disease surveillance systems but with Google Trends data. Strong correlations between RSV clinical surveillance data and Google Trends search results from several countries were observed. In monitoring an upcoming RSV outbreak with MEM, data collected from both systems yielded similar estimates of country-specific epidemic thresholds, starting time, and duration. We demonstrate in this study the potential of monitoring disease outbreaks in real time and complement classical disease surveillance systems by leveraging online search data.

## 1. Introduction

Respiratory syncytial virus (RSV) is the most common single cause of respiratory hospitalization of infants and is the second largest cause of lower respiratory infection mortality worldwide [1]. Currently there is no vaccine against RSV, although many preventive strategies are under development [2]. Historically, infection rates typically rise in late autumn and early winter in temperate climates. However, the seasonal patterns of several infectious diseases, including RSV, have been disrupted by the COVID-19 pandemic [3,4,5]. Specifically, RSV outbreaks were suppressed at the beginning of the COVID-19 pandemic (i.e., during the 2020–2021 period) [6] and resumed with irregular timing and increased magnitude from 2021–2022 onwards [7,8], e.g., see Japan [9], United States [10], United Kingdom [8], Turkey [11], Belgium [12] and Italy [13] in Northern Hemisphere, as well as Australia [14,15,16], New Zealand [17], Chile, Africa, Brazil [18] and other countries in the Southern Hemisphere [10]. These findings highlight the value of surveillance systems for RSV and other respiratory diseases during and after future pandemics, as the lifting of mitigation measures may result in severe outbreaks occurring with irregular timing [15]. Nevertheless, in most countries RSV is not a notifiable disease. Even in developed countries, surveillance systems have started monitoring RSV activity only recently. For example, New South Wales, Australia, only made RSV notifiable on 1 September 2022 [19].

Internet searches have previously been used to identify the timing, location, and magnitude of infectious disease outbreaks. In 2004, Johnson et al. reported a correlation between the frequency of influenza-related article access and the CDC’s surveillance data [20]. Similar results have been reported in Canada [21] and with Yahoo search queries [22]. In 2009, a study by Google and the CDC provided the first proof-of-concept for using Google search queries to detect influenza epidemics [23]. Since then, many influenza-related analyses have been conducted around the world, for example, in South Korea [24], Latin America [25], at a regional level in Manitoba, Canada [26] and South China, China [27], and at a city level in New York City [28] and Baltimore [29] in the US. Google Trends has also been used as a surveillance tool for other diseases, e.g., chicken pox [30], type 2 diabetes [31], dengue fever [32], Zika and Chikungunya [33], sexually transmitted infections [34], and COVID-19 [35].

Google Trends studies can be characterized into three main areas [36]: identifying seasonality, examining correlations between surveillance data and Google Trends, and forecasting. Seasonality has most commonly been investigated through visual observation [37,38,39,40,41,42], Kruskal–Wallis test [43,44,45], and Cosinor analysis [42,46,47,48,49]. The Pearson correlation coefficient is the principal statistical measure used to compare surveillance data to Google Trends [24,27,50,51,52,53,54,55,56,57,58,59,60,61,62,63,64]. Forecasting is the least studied area. Only nine out of 104 studies reviewed in a 2018 systematic review were focused on prediction forecasting [36], with methods comprised of statistical modeling [30], linear regression [23,34,65,66], cross-correlation [58], and time series analysis (e.g., ARIMA [67]).

However, Google Trends as a surveillance tool has not been broadly applied to RSV, despite it being the leading cause of hospitalization in infants in developed countries. Two previous studies identified a correlation between Google Trends and RSV [68,69], while another found that searches for RSV can predict pediatric RSV encounters [70]. All of these studies have been retrospective. To our knowledge, there have been no studies focused on the prospective use of Google Trends as a surveillance tool to forecast the emergence of RSV outbreaks.

Our study aims to rigorously leverage real-time, online search engine data to nowcast emergence of RSV and consequently better manage the contemporary uncertainties enhanced by the ongoing COVID-19 pandemic. In this paper, we (1) investigated the correlation between RSV clinical surveillance data and Google Trends data observed at both country and city levels, (2) compared epidemiologic estimates derived from the Moving Epidemic Method (MEM) over Google Trends and clinical surveillance data, (3) discussed the use of Google Trends as a surveillance tool to nowcast the emergence of RSV outbreaks for countries where RSV surveillance data are limited.

## 2. Materials and Methods

### 2.1. Data

Google Trends data [71] reflect how a specific search interest varies for a region over time. It ranges from 100% to 0%, scaled by the highest search number that a specific search interest ever generated within the chosen time period. Weekly or monthly data points are shown if the chosen time period is shorter or longer than 5 years, accordingly. In this study, Japan, Germany, and Belgium were selected for illustration purposes because of their high quality surveillance data and Google Trends data. Five full years of weekly clinical and Google Trends search data were included for each country in this study. Among many options, one relevant keyword which shows the clearest seasonal pattern for each country was selected: “RS Virus” for Japan, “RS Virus” for Germany, and “RSV” for Belgium and the other 14 countries. Weekly RSV case data were gathered from each country’s official open access website, including the National Institute of Infectious Diseases [72] for Japan, the Robert Koch Institut [73] for Germany, and the Belgian Institute for Health [74] for Belgium.

### 2.2. Correlation

The Pearson correlation coefficient has been commonly used to assess the correlation between Google Trends search data and clinical surveillance data [64]. In this study, a Python library package, SciPy [75], was applied to perform the correlation analysis.

### 2.3. The Moving Epidemic Method (MEM)

The World Health Organization (WHO) released a guideline on assessing the severity of influenza in seasonal epidemics and pandemics in 2017 [76] and suggested two types of thresholds to characterize the start of an epidemic, one based on the Moving Epidemic Method (MEM) [77,78] and the other based on the pre-selected weekly positivity rates. However, using a certain percentage of RSV positivity among all RSV tests in a season as a threshold to define an epidemic [79,80,81] does not provide insights into the intensity of the epidemic and should not be used prospectively to detect the start of an epidemic [82]. In comparison, the epidemic threshold generated by MEM provides a good balance between sensitivity and specificity to detect seasonal epidemics and to avoid false alerts from data noises [77]. MEM has been widely adopted to calculate epidemic thresholds for both influenza [77,78,83,84] and RSV [82]. There have also been studies applying the MEM to multiple indicators (outpatient visits, hospitalization, and mortality rate) and combining multiple thresholds to classify the severity of an influenza season [85]. However, to our knowledge, MEM has not been applied over Google Trends data as an early-warning system for infectious disease outbreaks.

MEM is modeled based on historical data from a specific country or region. The method has three main steps [77,78]. In the first step, each season is separated into three periods: pre-epidemic, epidemic, and post-epidemic period. For each season separately, the length of the epidemic period is determined by an optimization process that maximizes positive cases within the least number of consecutive weeks. In the second step, the epidemic threshold is calculated as the upper limit of the 95% one-sided confidence interval of the arithmetic mean of the 30 highest pre-epidemic weekly rates from all seasons. The number of highest rates from each season is 30/(number of seasons). This epidemic threshold defines the start of the epidemic. In the third step, medium, high, and very high intensity thresholds are calculated as the upper limits of the 40%, 90%, and 97.5% one-sided confidence intervals of the geometric mean of 30 highest epidemic weekly rates. For the purpose of sensing the start of the epidemic, the first 2 steps are sufficient. The third step is to estimate the intensity of an epidemic. By dividing each season into three periods, the epidemic threshold is calculated only based on data points within the epidemic period, excluding false alerts of those abnormal high weekly rates during the pre-epidemic periods. By comparing the current week’s value against the epidemic threshold, we can know if the country being investigated is experiencing an epidemic period.

We first applied the MEM over countries with both clinical surveillance and Google Trends search data. The consistency of epidemiologic estimates derived from MEM with clinical surveillance vs. Google Trends search data were investigated to validate whether Google Trends can represent the clinical surveillance data in terms of estimating the epidemic starting week and duration. The ‘mem’ library in R was used in this study [86]. Google Trends search data applied with MEM were prepreprocessed with Loess transformation with default fixed criterium method provided in ‘R-mem’ library.

We also applied MEM to countries with limited publicly accessible clinical surveillance data. MEM thresholds estimated over the Google Trends data can be interpreted whether they are reliable or not without directly comparing to clinical case data. To interpret the reliability of the results, we can examine the goodness of fit of the MEM model using estimators such as sensitivity, specificity, positive predictive value, percent agreement, Matthews correlation coefficient, etc. In this paper, the Matthews correlation coefficient is reported. Details about how goodness of MEM is estimated are explained in [86]. Besides the goodness of fit, the epidemic percentage, which is the proportion of cases in the epidemic period over all cases, is generally a good indicator for understanding if there is a clear seasonality pattern and how well the MEM performed.

## 3. Results

### 3.1. Identical Seasonal Patterns between Google Trends and Case Data

**Figure 1:**
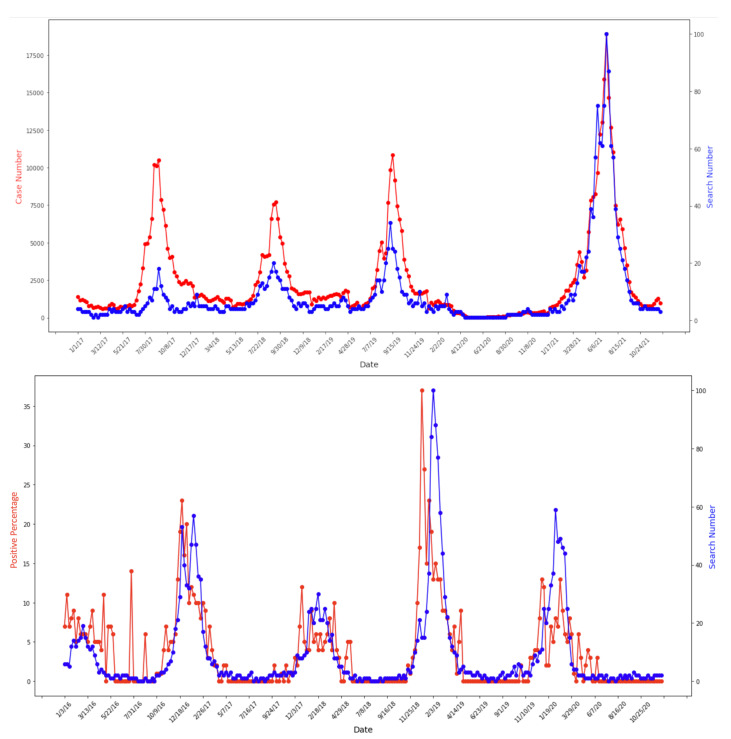
Correlation between Google Trends and case data at country level: Japan 2017–2021 (**top**) Pearson correlation coefficient = 0.87, *p*-value < 0.0001. Germany 2016–2020 (**bottom**) Pearson correlation coefficient = 0.65, *p*-value < 0.0001. Google Trends and case data are marked in blue and red accordingly. Y-axis for both Google Trends and case data ranges from the minimum to the maximum values of each function over time.

**Figure 2:**
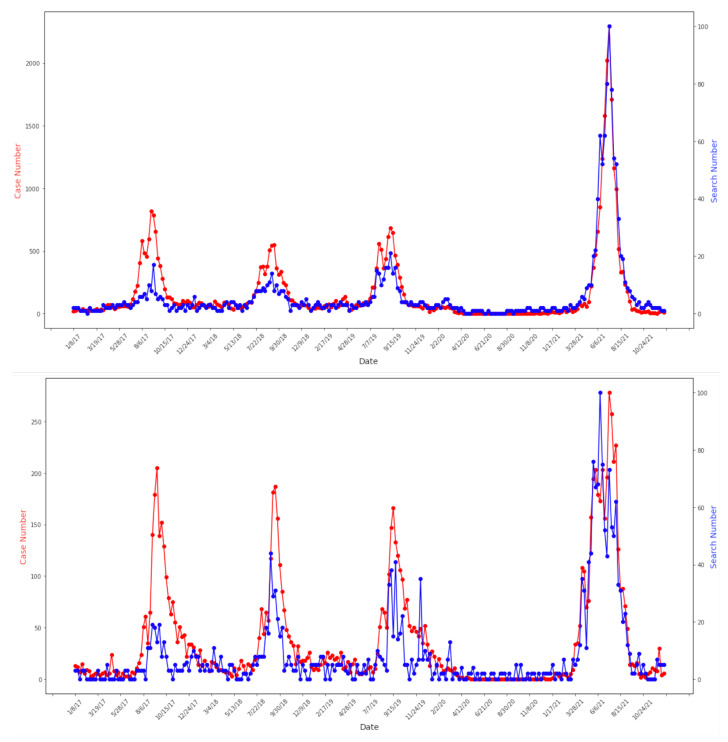
Correlation between Google Trends and case data at regional level: Tokyo 2017–2021 (**top**) Pearson correlation coefficient = 0.92. Kyoto 2017–2021 (**bottom**) Pearson correlation coefficient = 0.83. Both *p*-value < 0.0001. Google Trends and case data are marked in blue and red accordingly. Y-axis for both Google Trends and case data ranges from the minimum to the maximum values of each function over time.

Google Trends data matched case data with no delay in terms of seasonal start time, end time, and peak time for each epidemic in Japan and Germany (with Pearson correlation coefficient = 0.87, *p*-value < 0.0001 for Japan and Pearson correlation coefficient = 0.65, *p*-value < 0.0001 for Germany) (Figure 1). Note that weekly reports from Japan contained absolute case numbers from all sentinel hospitals, while reports from Germany contained positive test rates, where the test sample sizes ranged throughout the year. As a result, the correlation between case and search data for Germany was lower due to a higher fluctuation of cases. We did not apply any smoothing preprocessing on any of the data. These matched patterns were also found at the regional level (Figure 2). Tokyo and Kyoto analyses are presented here to illustrate this point.

**Figure 3:**
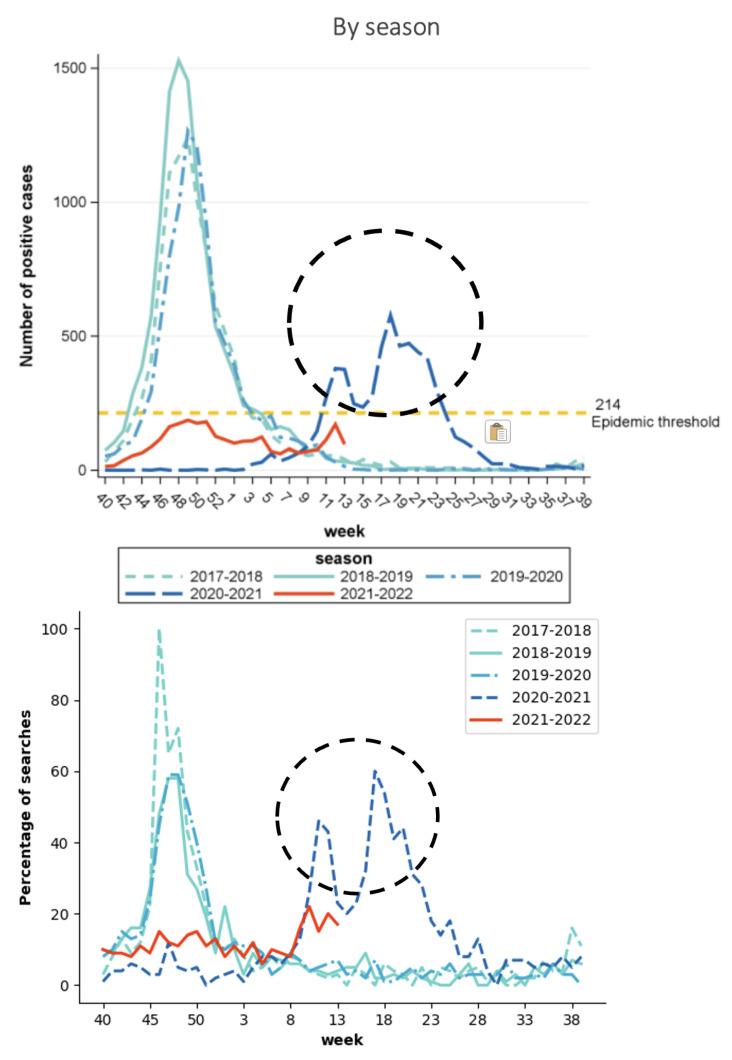
RSV seasonality for Belgium: identical patterns within each season (i.e., bimodal for season 2020–2021, unimodal for other seasons) observed in both case (**top**) and Google Trends (**bottom**) data.

Google Trends data were also able to capture intra-annual abnormalities observed in case data. Using Belgium as an illustration, the seasonal patterns of RSV outbreaks can be seen in both case and search data shown in Figure 3. For the 2020–2021 season, the outbreak started later than previous years and had two peaks. This was also observed in the Google Trends data (shown above in Figure 3).

### 3.2. Identical Epidemiological Estimates from Case and Google Trends Data

Identical epidemic estimates in terms of season starting week and duration were obtained by applying MEM to Google Trends and clinical case data. In the cases of Japan and Germany, MEM provided identical estimates for the average start week and epidemic duration from the clinical case and Google Trends search data, with detailed results shown in Table 1.

### 3.3. Epidemic Estimates from Google Trends Data in Countries with Limited Case Surveillance

**Figure 4:**
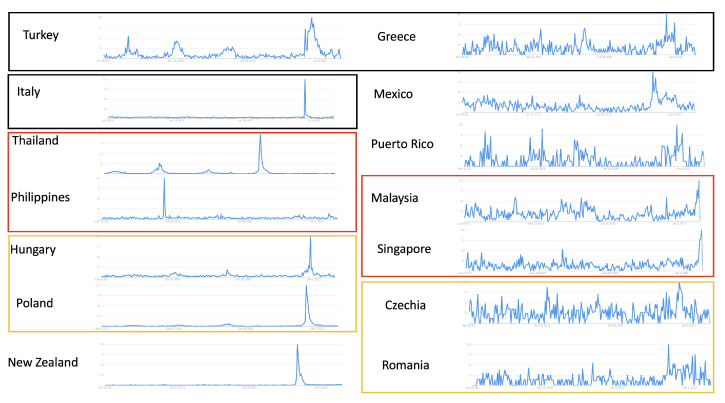
Google Trends of countries with limited surveillance: using the same keyword “RSV”, countries with clear patterns are listed on the (**left**). Countries without clear patterns are listed on the (**right**). Countries that are close to each other geographically are boxed in the same color.

We selected 14 countries without RSV clinical surveillance data to investigate using Google Trends. Using the same keyword “RSV”, seven out of 14 countries selected showed clear visual seasonal patterns (left panel of Figure 4) and the rest did not (right panel of Figure 4). Countries with clear visual seasonal patterns in Figure 4 generally correspond to higher epidemic percentage values. The purpose of using the same keyword is to illustrate how we can interpret whether the data with the chosen keyword is reliable or if the keyword is good enough to capture the pattern.

We then applied MEM to Google Trends data for the selected 14 countries to generate insightful epidemiologic estimates (Table 2). The surveillance column shows whether the countries are experiencing an epidemic based on MEM estimates. Current values for week 25, 2022 and thresholds were calculated based on the data of the five years before week 25, 2022, excluding 2020 and 2021 (due to abnormal RSV activities compared to other years) using MEM. A country is considered to be entering an epidemic if its current Google trend data value is above the estimated epidemic threshold dynamically generated by MEM. The column of 2020–2021 in Table 2 shows the start week and end week of last season. We are aware that the COVID-19 pandemic shifted the starting time for RSV outbreak in some countries. The 2020–2021 column is listed for a comparison to investigate whether this phenomenon can also be observed over the Google Trends data. Countries with clear visual seasonal patterns in terms of fewer fluctuations in Figure 4 on the left generally correspond to higher epidemic percentage values (Table 2 on the top), with a few countries as exceptions. As an unusually high RSV peak for Italy, the Philippines, Hungary, Thailand, Poland and New Zealand can dominate all other seasonal peaks and patterns in Google Trends, we attempted a few data selection heuristics to improve the method’s fitting results. One choice is to heuristically exclude abnormal, one-time-only epidemic peak data. For example, excluding Google Trends data for 2020 and 2021 for Hungary, Poland, Thailand and New Zealand resulted in an improved fitting performance (evaluated by the epidemic percentage value). By contrast, the low fitting performances for the Philippines and Italy have not yet been resolved, partially due to the facts that the abnormal peak is outside 2020 and 2021 and the identified Google Search data has an authentic unclear seasonal pattern, respectively.

## 4. Discussion

Google Trends can complement existing surveillance systems for monitoring disease outbreaks in real time. Using RSV as a case study, we revealed the strong correlation between Google Trends and clinical case data from Japan and Germany. We also observed that although many countries generate high quality case data, weekly reports may be delayed for several weeks due to various reasons. Google Trends can be used as a supplemental surveillance system for countries with limited sentinel network coverage, as well.

Google Trends is also not linked to the number of sentinel hospitals or the variation in reporting between testing sites. Most countries may not be able to extend their surveillance systems to collect data from all hospitals on time or maintain a reliable testing sample size across different times of the year. The positive testing rates may be sensitive to the testing sample size, creating false alarms as a result.

In our multiple country comparison effort, we also observed that Google Trends data were of a higher quality among countries with better surveillance systems. This may be due to socioeconomic factors such as better public health education that drove information seeking behavior online.

Occasionally, a single keyword such as “RSV” or “RS Virus” could be sufficient for identifying the clear seasonality patterns for RSV in Google Trends in certain countries, but not all: each country’s most suitable keyword for monitoring RSV outbreaks is still highly dependent on the local language choice. Unlike flu, adding additional keywords describing the disease symptoms may weaken the patterns, as many respiratory pathogens share a common pool of flu-like symptoms. Additionally, preprocessing the search data and then using MEM could prevent the false alarms caused by noisy fluctuation in the trends.

However, there can be issues with Google Trends. For example, Google Trends data are scaled based on the highest value in the time frame of choice. The abnormally high volume of Google Trends searches in 2020–2021 due to the COVID-19 pandemic scales down the rest of the normal seasons, which diminishes their seasonality patterns. The peak in 2020–2021 caused the epidemic threshold to considerably shift up compared to previous years. Therefore, when applying MEM to estimate country-specific outbreak thresholds, we excluded data from 2020 to 2021 because travel restrictions were in place in most countries. However, when obtaining data from Google Trends, 2020–2021 data were included to keep the current data point on the same scale as previous years, since Google Trends cannot exclude certain years. Additionally, monthly instead of weekly Google Trends data will be displayed if the time period selected on the platform is specified to be longer than five years. When there are no clinical case data to compare Google Trends data against, we can examine the epidemic percentage as an indicator of how the MEM performed. If the epidemic percentage is low, the estimates from MEM from Google Trends data may not be reliable. For example, although Italy had clear seasonal Google Trends patterns, searches at the start of the COVID-19 pandemic diminished compared to previous years, leaving the epidemic percentage value low and unreliable. One possible solution may be referring to the results from nearby countries in the same geographic region (Figure 4).

Notably, when the disease is not that well known (such as RSV), people tend to search for multiple keywords. Since respiratory diseases share similar symptoms, it may be challenging to collect accurate keywords for a particular disease or identify a seasonal pattern specific caused by a specific disease.

Additionally, both lower search volumes and clinical cases were observed after vaccination was introduced for other diseases such as rotavirus [87]. It remains unclear how much this would affect the correlation between clinical case and Google Trends search data for RSV, or if predicting using Google Trends data remains as sensitive as before a vaccine was introduced.

## 5. Conclusions

Google Trends can complement existing surveillance systems to monitor disease outbreaks in real time, especially in countries with limited or no sentinel network surveillance. Search data correlated well with clinical case data when both were available. Identical estimates of epidemic start time and duration were obtained from MEM using both Google Trends and clinical case data. The quality of clinical case data from countries with surveillance systems is linked to the sentinel hospital surveillance systems. This further identifies the importance of using alternative data streams, such as internet search data, to assist in locations where surveillance systems are not well established.

## Figures and Tables

**Table 1:** Epidemic estimates of Japan 2015–2020 using MEM: identical epidemic seasonal start time and length were obtained by applying MEM over Google Trends and case data.

	Japan		Germany	
Data Source	Case	Google Trends	Case	Google Trends
Goodness (Matthews correlation coefficient)	0.75	0.64	0.44	0.83
Epidemic percentage	61.03%	54.76%	71.95%	75.28%
Average start week	33	33	1	1
Average length	14	14	12	12

**Table 2:** MEM estimates for countries with limited surveillance for week 25, 2022: Estimates were based on Google Trends data between week 25, 2022 and previous 5 years, excluding 2020 and 2021 due to their abnormal intensified RSV activities. The upper part of the table contains countries listed on the left in Figure 4 with clear patterns. The lower part of the table contains countries listed on the right in Figure 4 with no clear patterns. Both parts are ranked by epidemic percentage, which is the ratio of cases during the epidemic to all cases.

	Estimator				Surveillance			2020–2021	
Country	Epidemic Percentage	Start Week	Aver. Length	Epidemic Threshold	Current (W25, 2022)	Above Threshold?	For How Long (Week)?	Start Week	End Week
Poland	72.75	1	15	1.26	1.20	NO		38	50
Thailand	62.33	31	15	4.02	1.06	NO		47	4
Turkey	55.06	49	14	13.77	12.59	NO		40	1
New Zealand	52.21	31	11	0.22	2.24	YES		11	36
Hungary	44.25	6	12	4.46	3.51	NO		44	4
Philippines	27.19	45	9	5.0	2.79	NO		33	37
Italy	2.4	13	1	3.89	4.32	YES		40	51
Puerto Rico	59.59	44	13	22.02	28.60	YES	+1	45	2
Greece	34.97	2	10	23.93	10.52	NO		42	1
Malaysia	22.19	33	7	40.45	79.61	YES	+6	19	now
Romania	15.11	4	5	17.25	13.08	NO		35	35
Singapore	9.27	26	3	21.12	88.83	YES	+5	20	now
Czechia	8.5	5	3	36.16	27.30	NO		42	50
Mexico	2.82	26	1	15.33	19.40	NO		31	37

## Data Availability

Not applicable.

## Abbreviations

The following abbreviations are used in this manuscript:

COVID-19	Coronavirus disease
RSV	Respiratory syncytial virus
MEM	Moving Epidemic Method
